# Medial-tonsillar telovelar approach for resection of a superior medullary velum cerebral cavernous malformation: anatomical and tractography study of the surgical approach and functional implications

**DOI:** 10.1007/s00701-020-04418-2

**Published:** 2020-06-10

**Authors:** Christian Brogna, José Pedro Lavrador, Hussein Shaaban Kandeel, Ahmad Beyh, Eduardo C. Ribas, Francesco Vergani, Christos M. Tolias

**Affiliations:** 1 Department of Neurosurgery, King’s College Hospital, Denmark Hill, London, SE5 9RS UK; 2 Basic and Clinical Neuroscience, Institute of Psychiatry, Psychology and Neuroscience, King’s College London, London, UK; 3 NatBrainLab, Centre for Neuroimaging Sciences, Institute of Psychiatry, Psychology and Neuroscience, King’s College London, London, UK; 4 Division of Neurosurgery, Hospital das Clínicas, University of São Paulo Medical School, São Paulo, Brazil; 5 Albert Einstein Hospital, São Paulo, Brazil

**Keywords:** Cerebral cavernous malformation, Superior medullary velum, Superior cerebellar peduncle, Tractography

## Abstract

**Background:**

Superior medullary velum cerebral cavernous malformations pose a challenge in terms of appropriate microsurgical approach. Safe access to this deep location as well as preservation of surrounding anatomical structures, in particular the superior cerebellar peduncle just lateral to the superior medullary velum and the dentate nuclei, is paramount to achieve a good functional outcome.

**Methods:**

Cadaveric dissections provide useful knowledge of the normal anatomy while tractography allows a better understanding of the individual anatomy in the presence of a lesion. The medial-tonsillar telovelar approach provides a feasible corridor for accessing superior velum cerebral cavernous malformations without compromising the fibres contained in the superior cerebellar peduncle. The major cerebellar efferents—cerebello-rubral, cerebello-thalamic and cerebello-vestibular tracts—and afferents, anterior spinocerebellar, tectocerebellar and trigeminocerebellar tracts, within the superior cerebellar peduncle are preserved, and the dentate nuclei are not affected.

**Results and conclusion:**

A retraction-free exposure through this natural posterior fossa corridor allows the patient with the anatomical and functional subtract to make a good functional recovery by minimizing the risk of a superior cerebellar syndrome, ataxia, tremor and dysmetria; decomposition of movement in the ipsilateral extremities, nystagmus and hypotonia; or akinetic mutism, reduced or absent speech with onset within the first post-operative week.

## Introduction

Cerebral cavernous malformations of the superior medullary velum represent a challenge in terms of safest surgical approach. A very good understanding of the anatomical structures surrounding the superior medullary velum and the efferent pathways of the cerebellum at risk of being damaged is mandatory to approach the superior medullary velum region. Moreover, access to this deep region requires knowledge on how to enhance the advantages offered by opening microsurgically the uvulotonsillar compartment of the cerebello-medullary fissure and consequently the tela choroidea and the inferior medullary velum to gain access to the cavernous of the superior medullary velum.

## Material and methods

### Relevant microsurgical anatomy

The *cerebellomedullary fissure* separates the cerebellum from the medulla, extending upwards between these regions. This fissure is at the dorsolateral aspect of the inferior half of the roof of the fourth ventricle and has to be surgically enlarged in order to perform the telovelar approach. Its anterior wall is formed by the posterior surface of the medulla, the inferior medullary velum and the tela choroidea; its posterior wall is formed by the uvula and nodule of the vermis medially and the tonsils and biventral lobules laterally [22]. (Fig. 3d).

The *inferior medullary velum* is a thin bilateral semitranslucent butterfly-shaped sheet of neural tissue that stretches from the nodule medially and to the flocculus laterally at each side. It is attached inferiorly to the tela choroidea at the telovelar junction. (Fig. 3d) It is continuous with the superior medullary velum at the level of the fastigium.

**Fig. 1:**
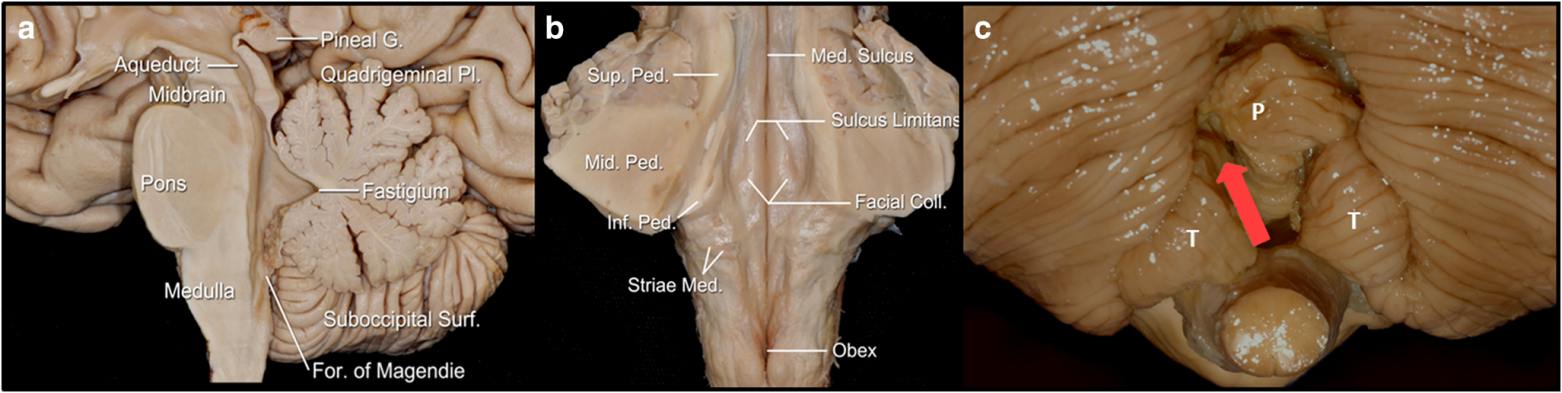
Main anatomic external landmarks of brainstem and cerebellum: **a** Midline sagittal view of the brainstem and cerebellum; **b** coronal view at the level of the middle cerebellar peduncles of the floor of the fourth ventricle; **c** view of the suboccipital surface of the cerebellum (P, Pyramid; T, Tonsil) showing the natural corridor used in the unilateral uvulotonsillar approach (red arrow)

**Fig. 2:**
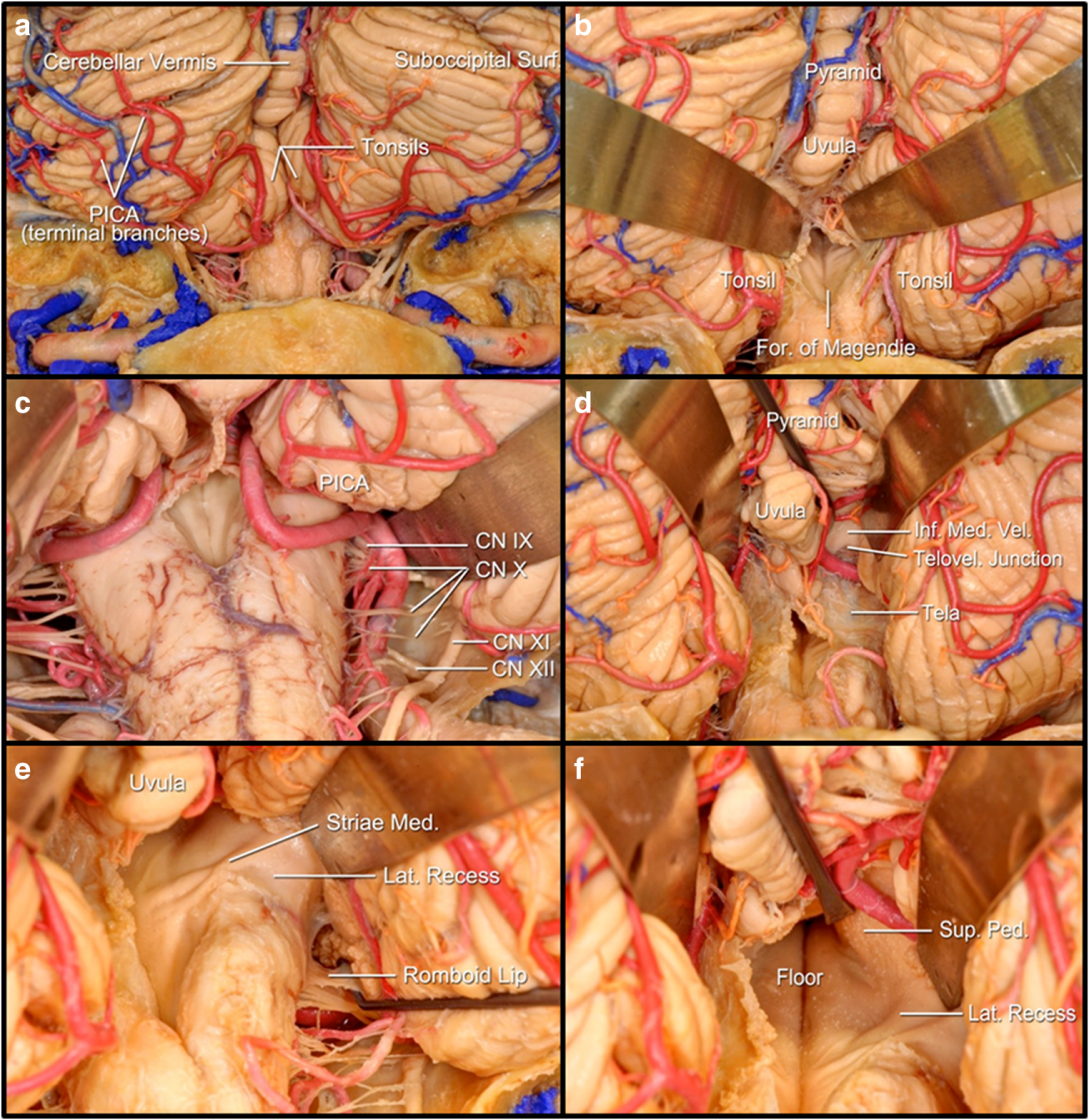
Anatomy of the microsurgical approach to the superior medullary velum via a medial-tonsillar approach: **a** suboccipital surface of the cerebellum and posterior aspect of the medulla oblongata; **b** brain retractors on top of the tonsils to expose the corridor in-between the tonsils (laterally) and the uvula and pyramid (medially) with exposure of the foramen of Magendie; **c** posterior inferior cerebellar artery (PICA) trajectory from the lateral aspect of the medulla oblongata (with its relation with the IX, X, XI and XII cranial nerves) towards the cerebellar-medullary fissure and its relation with the tonsil and the uvula; **d** exposure of the tela choroidea, inferior medullary velum and the telovelar junction; **e** exposure of the floor and lateral recess of the fourth ventricle after dividing the tela choroidea and the inferior medullary velum; **f** exposure of the superior cerebellar peduncle above the lateral recess of the fourth ventricle

The *tela choroidea* forms the lower portion of the inferior half of the roof fourth ventricle and extends from the nodule medially to the lateral recess laterally. The tela choroidea does not completely enclose the forth ventricle and forms three openings which connect this cavity to the subarachnoid space: the paired foramina of Luschka, located at the outer margins of the lateral recesses, and the foramen of Magendie, located just inferior to the uvula [19]. Caudally, the tela choroidea includes the inferolateral edges of the floor along narrow white ridges, the teenier, which meet at the obex (Figs. 1 and 2).

The *posterior inferior cerebellar artery* (PICA) originates at the vertebral artery and course within the cerebellomedullary fissure, forming its telovelotonsillar segment between the tonsil posteriorly and the tela choroidea and inferior medullary velum anteriorly (Fig. 2c). It loops around the superior pole of the tonsil, forming a convex curve referred as the supratonsillar loop, where it faces the inferior medullary velum. The PICA is responsible for supplying the inferior medullary velum, the inferior cerebellar peduncle and the suboccipital cerebellar surface [12].

The *superior cerebellar peduncle* (SCP) is the largest cerebellar efferent bundle, which is a group of fibres that emerge from the hilus of the dentate nucleus. These fibres pass rostrally into the upper fourth ventricle. The superior cerebellar peduncles emerge from the upper and medial part of the white substance of the hemispheres. They project in parallel and are placed under cover of the superior and inferior colliculi. They connect the dentate nucleus to the red nucleus—where they decussate, forming the dentatorubrothalamic tract, part of Guillain-Mollaret triangle (with the connection in between the inferior olivary nucleus with the red nucleus via the central tegmental tract and the dentate afferents from the same inferior olivary nucleus). The superior cerebellar peduncles are joined by the superior medullary velum, can be followed up to the inferior colliculi and disappear in the red nucleus (Figs. 1 and 2f). A comprehensive description of all the tracts passing through the SCP is provided in Table 1.

**Table 1:** Superior cerebellar afferents and efferent tracts

Superior cerebellar peduncle		
Quality of the fibres	Tracts	Information
Afferent fibres	Anterior spinocerebellar tract	Proprioceptive information form the ipsilateral lower limb
	Tectocerebellar tract	Visual and auditory stimuli
	Trigeminocerebellar tract	Proprioceptive information from the face
Efferent fibres	Cerebello-rubral tract	Regulation of muscular tonus on the contralateral side
	Cerebello-thalamic tracts	Regulation of muscular tonus on the contralateral side
	Cerebello-vestibullar tract	Balance regulation

The *superior medullary velum* (valve of Vieussens; anterior medullary velum) is a thin lamina of white matter extending between the cerebellar peduncles and participates in the formation of the roof of the fourth ventricle. The dorsal surface of the lower half is covered by the lingula of the cerebellum, caudal to the exit of the fourth cranial nerve, and separating the lingula from the superior medullary velum creates a working corridor (the cerebellomesencephalic fissure) [22]. The superior medullary velum does not appear to be a uniform structure when one moves from a mediolateral direction as it forms a larger angle with the floor. It connects both superior cerebellar peduncles. Besides the decussating fibres of these cerebellar peduncles which lie deep in relation to the superior medullary velum, there are few essential neural structures in the composition of the superior medullary velum, making its sacrifice, not clinically significant, especially caudal to the decussation of the trochlear nerves [30] (Fig. 2a). The superior medullary velum receives its arterial supply from the superior cerebellar artery via its precerebellar branch [23]. Venous drainage is by the superior cerebellar peduncle veins, which unite superiorly at the emergence of the trochlear nerve from the brainstem, to form the vein of the cerebellomesencephalic fissure [24].

The *uvulotonsillar telovelar approach* is developed in between the structures of the paleocerebellum, mainly responsible for the muscle tonus. The medial tonsillar surface is displaced laterally away uvula, exposes the inferior half of the ventricular roof (tela choroidea and inferior medullary velum) and the opening the tela choroidea upward to the telovelar junction provides access to the floor of the fourth ventricle, from the aqueduct to the obex and to the medial portion of the lateral recesses [25] (Fig. 2c–f). The junction of the uvula and pyramid limits the lateral and medial retractions of the tonsil and the uvula at the uvulotonsillar space, limiting the exposure of the cisternal surface of the superior half of the ventricular roof. However, the inferior medullary velum is continuous at the level of the fastigium with the superior medullary velum, and the additional opening of the inferior medullary velum can improve the exposure of the ventricle roof, including the fastigium, superolateral recesses and the ventricular surface of the superior half of the roof [25] (Fig. 2c–f).

### Diffusion MRI data and tractography dissection

Diffusion MRI data were obtained for one healthy young adult (male, age category 26–30 years) from the Human Connectome Project (Van Essen et al., 2013) (https://www.humanconnectome.org/) [32]. The data were acquired on a 3T Siemens “Connectome Skyra” scanner using a 32-channel head coil and consisted of 90 diffusion directions; *b* value = 2000 s/mm2; 18 B0 volumes; 1.25 mm isotropic voxels; TE = 89.5 ms and TR = 5520 ms. Data were corrected for motion, eddy-current distortions and susceptibility distortions, according to the HCP minimal pre-processing pipelines [14]. Diffusion tensor (DTI) modelling and tractography were performed in StarTrack (https://www.mr-startrack.com/) with a fractional anisotropy (FA) threshold of 0.2, step size of 0.5 mm and angle threshold of 30°.

**Fig. 3:**
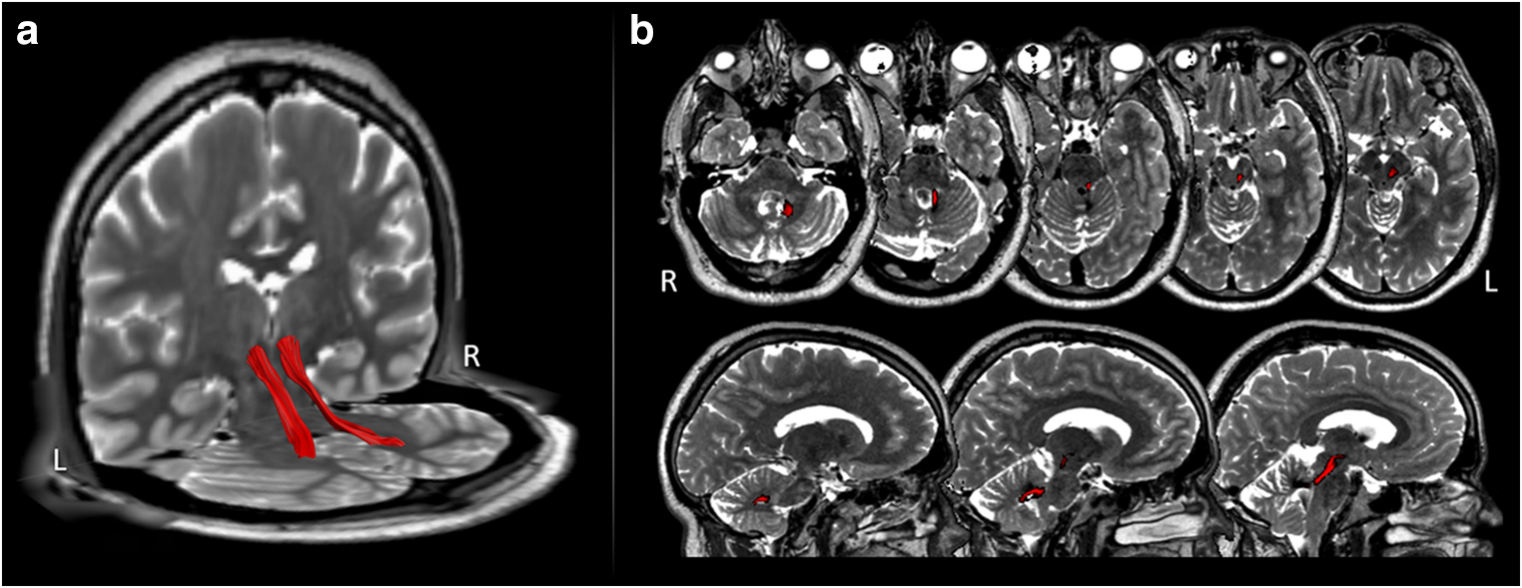
Dentatorubrothalamic tract dissection: 3D (**a**) and 2D (**b**) dissection of the dentatorubrothalamic tracts (red) imposed on the patient’s T2 volumetric MRI

The superior cerebellar peduncles were manually dissected in TrackVis (http://trackvis.org/) based on sphere regions of interest (ROI). The first ROI was placed in the cerebellum at the level of the dentate nucleus and the second in the brain stem at the level of the red nucleus. Only tractography streamlines terminating in both ROIs survived the filtering process, thereby effectively isolating the pathway of interest. To visualize the control tractography data on the patient’s brain, linear and non-linear image registration were performed using the Advanced Normalization Tools [2]. Image registration was based on the T2w images as they provide good contrast in subcortical regions involving the dentate nucleus and red nucleus. The resulting affine matrix and warp field were applied to the density map of the control subject’s tracts. (Fig. 3).

## Case presentation

**Fig. 4:**
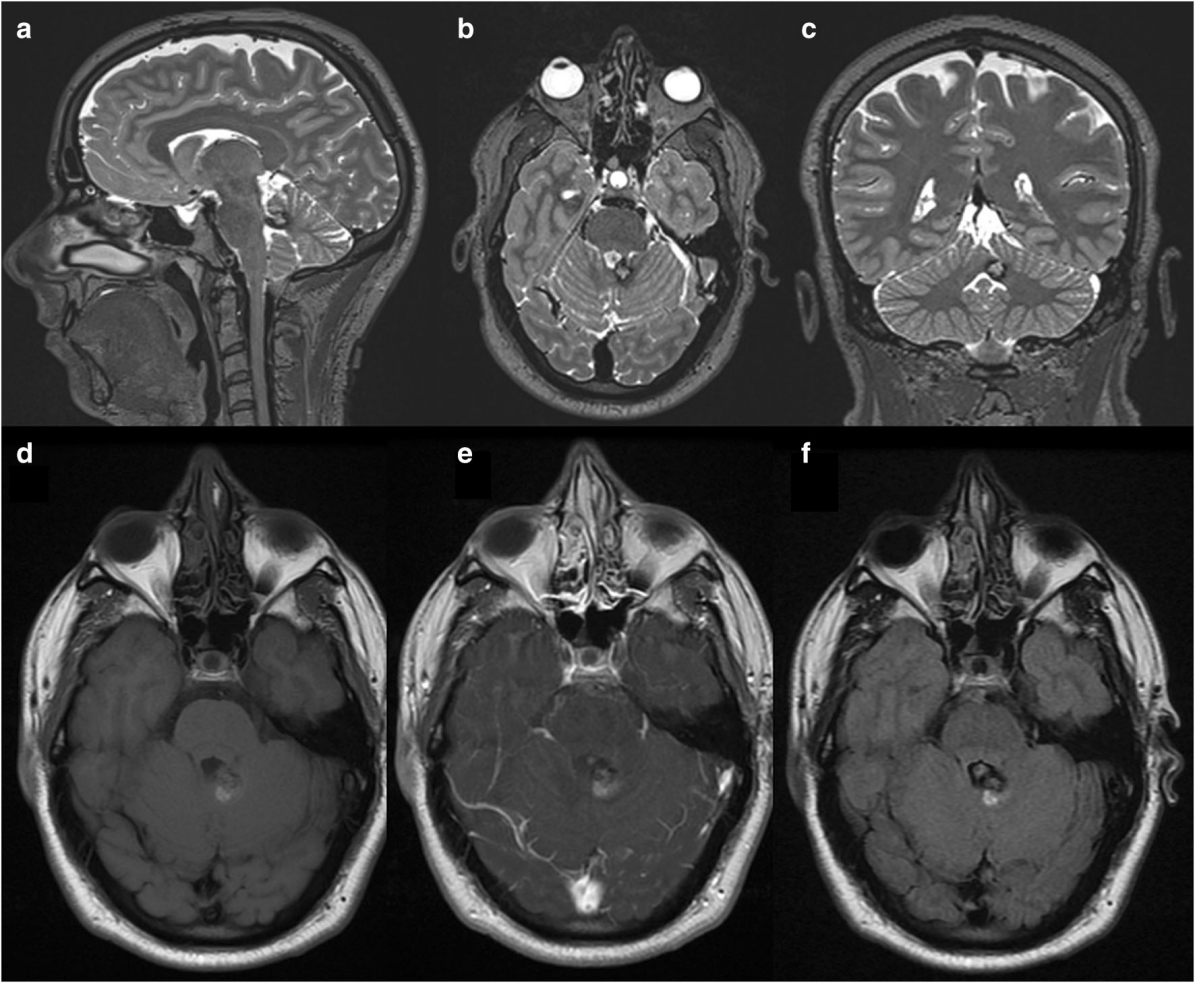
Pre-operative MRI: **a**–**c** T2-weighted images revealing the cerebral cavernous malformation on the superior medullary velum with a hypointense ring (Zabramsky type I). T1-weighted images—non-contrast (**d**) and with gadolinium (**e**)—and FLAIR (**f**) supporting acute blood around the cerebral cavernous malformation lesion

A 46-year-old man presented with a progressively deteriorating 1-year history of cerebellar ataxia and left superior arm intentional tremor. An MRI scan showed a round lesion in the superior medullary velum suggestive of a cerebral cavernous malformation. (Fig. 4) The patient had a right unilateral uvulotonsillar telovelar approach and successful resection of the lesion, proved to be cerebral cavernous malformation after histopathology report. Post-operative course was uneventful. At 3 months follow-up, the patient experienced a complete remission of symptoms, and he is currently under regular follow-up.

### Uvulotonsillar telovelar approach to the superior medullary velum

**Fig. 5:**
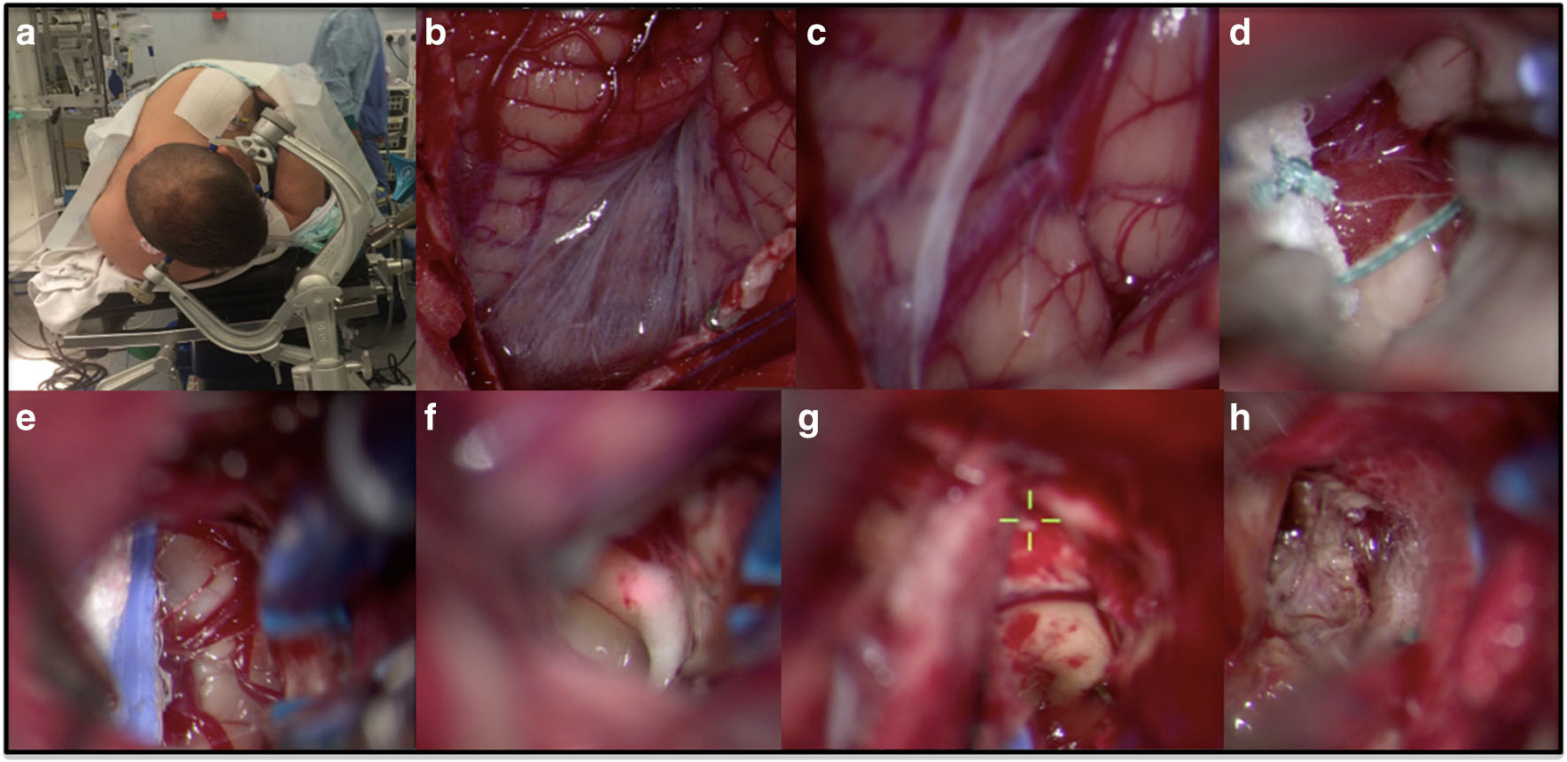
Surgical procedure: **a** posterior fossa approach; **b** and **c** cisterna magna opening and exposure of the posterolateral fissure; **d** PICA comes into view, tela choroidea and choroid plexus exposure; **f** inferior medullary velum exposed after dissecting the superior portion of the posterolateral fissure; **f** visualization of the aqueduct of Sylvius; **j** visualization of the superior medullary velum (green target); **k** resection of the superior medullary velum cerebral cavernous malformation

The patient was placed in pins in the park-bench position. A prone *Concorde* position or a semisitting position is also suitable alternatives. A posterior midline incision and a midline suboccipital craniotomy were performed. The superior aspect of C1 was drilled up to 1 cm off midline in order to gain the steeper angle required for the approach (the arch of C1 did not have to be completely removed in this particular case). The dura was opened under the microscope in a Y-shaped fashion. The arachnoid of the cistern magna was incised longitudinally and suspended to the dura with titanium Ligaclips (Ethicon®). With microneurosurgical technique, the uvulotonsillar compartment was carefully dissected and enlarged throughout its whole extension exposing the PICA, the tela choroidea and the inferior medullary velum. Fixed retraction to cerebellar structures was never applied through the whole procedure. The tela choroidea was opened starting from the foramen of Magendie and extending upward to the telovelar junction. Additionally, the inferior medullary velum was incised, and direct access was obtained to the upper portion of the fourth ventricle. Once the aqueduct of Sylvius was visualized inside the fourth ventricle for orientation, yellowish discoloration of the superior medullary velum was noted. Incision of the superior medullary velum 2 mm off midline on the right side allowed visualization of the cerebral cavernous malformation and its surrounding haemorrhagic constituents. The lesion was gently dissected and removed. No attempt was made to address the surrounding haemosiderin deposits in order to preserve the fibres of the superior cerebellar peduncle, in particular the dentatorubrothalamic fibres. Moreover, the superior medullary velum does not represent an epileptogenic zone, which would warrant removal of surrounding brain tissue with haemosiderin deposits (Fig. 5 and Surgical Video).

**Fig. 6:**
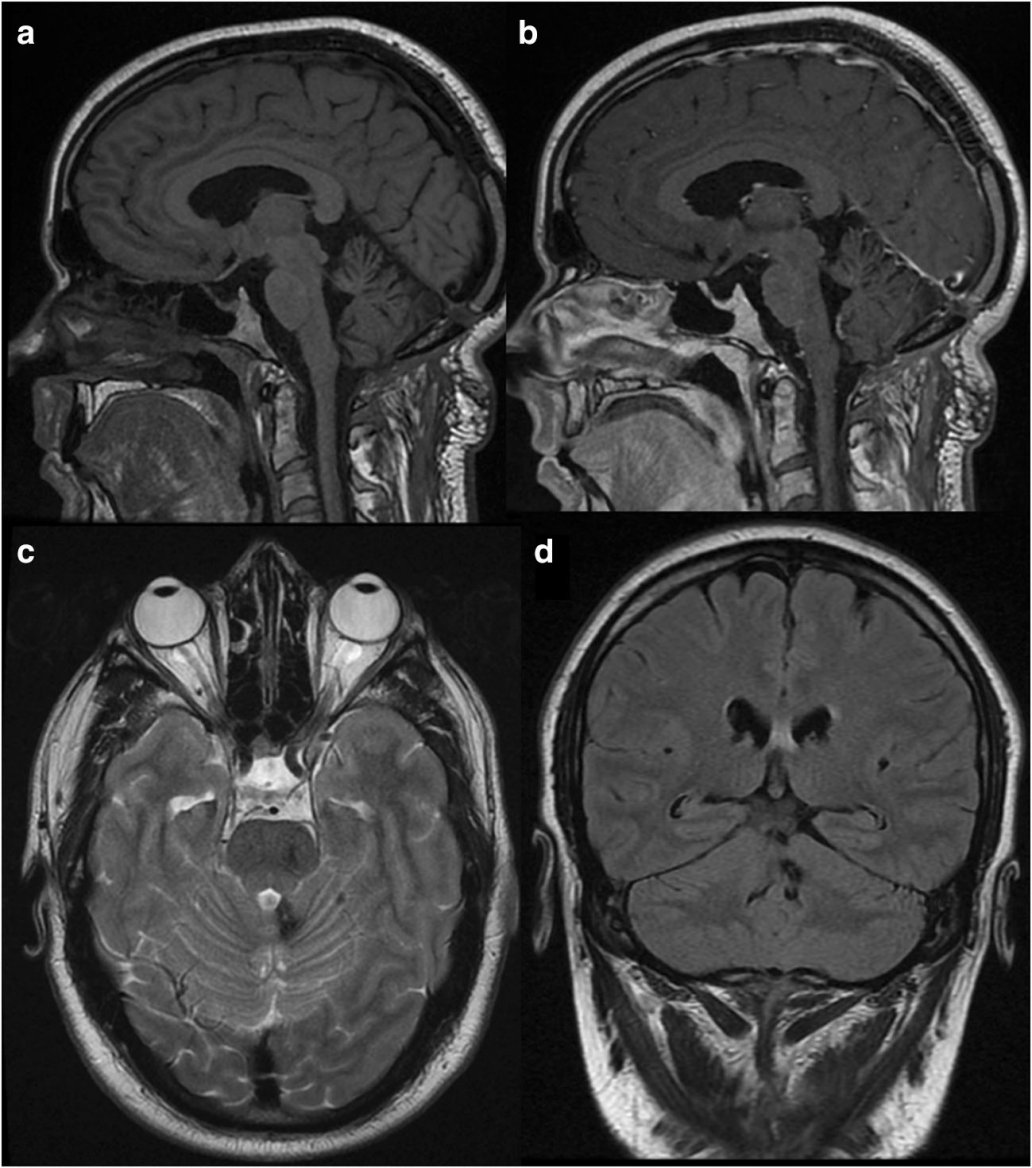
Post-operative MRI: T1-weighted images—non-contrast (**a**) and with gadolinium (**b**)—document absence of residual disease or complications related with the surgical resection. T2 weighted (**c**) and FLAIR (**d**) show the residual haemosiderin ring collapsed into the surgical cavity

The post-operative MRI documented complete resection of the cerebral cavernous malformation (Fig. 6).

## Discussion

Superior cerebellar peduncle syndrome was first described in monkeys by Ferrier and Turner in 1894 [11]. Other groups replicated their experiments in monkeys (Keller and Hare, 1934 [16]; Ferraro and Barrera, 1936 [10]; Walker and Boterrel, 1937 [33]). The main consequences of the complete surgical lesion of the SCP were ataxia (improved with time but do not resolve completely), tremor, dysmetria and decomposition of movement in the ipsilateral extremities. Nystagmus and hypotonia were temporary and not reproducible in all cases. The incomplete lesion of the SCP produced a mild and temporary syndrome.

Girard et al published in 1950 [13] the first description of this syndrome in humans in a context of superior cerebellar artery infarct. The link between the SCP and palatal myoclonus was initially performed by Trelles (1935) [29] and posteriorly described by the work performed by Lapresle and Ben Hamida (1965–1971) [17] who showed the pathway between the dentate nucleus, contralateral inferior olive and the red nucleus involving the SCP, the inferior cerebellar peduncle and the central tegmental tract. The hypertrophic olivary degeneration is a rare but well-known MRI sign of transsynaptic degeneration apparent 3–4 weeks after injury of Guillain-Mollaret triangle. Cerebral cavernous malformations and posterior fossa tumours are among the most common lesions in the neurosurgical literature [21].

Great attention has been given to the SCP in the last decade as a major player in the cerebellar mutism. This condition is characterized by reduced or entirely absent speech with onset generally within the first post-operative week, and multiple case reports in both children [3, 5, 20] and adult [31] with supporting DTI studies have documented unilateral atrophy of the SCP in relation with the cerebellar mutism.

**Fig. 7:**
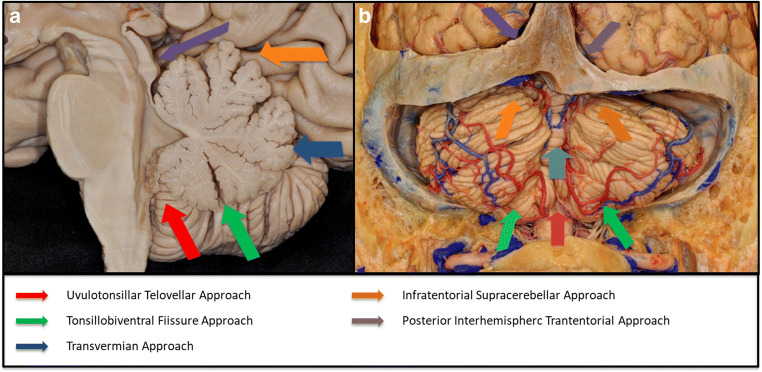
Schematic representation of the different surgical approaches to the superior medullary velum

These symptoms related SCP injury render crucial the preservation of this structure during the surgical approaches to SMV lesions. Both this function-preserving approach and the fact that the SCP is not known to be epileptogenic supported the decision of not removing the haemosiderin deposits around the lesion. This is supported by the most recent literature that advocates a careful risk-benefit approach to the haemosiderin deposits in eloquent cerebral cavernous malformations [8]. The classic surgical corridors are hampered by natural anatomic barriers: the cerebellum and the tentorium. When considering a space occupying lesion that enlarges and displaces the boundaries of the SMV—as a tumour—the growth pattern may facilitate a particular surgical approach [28]. However, the surgical approach for a lesion that grows within the SMV without enlarging it is difficult and hazardous when considering the corridor with the least anatomical disruption despite proper exposure for resection. The most common approaches are summarized in Fig. 7 and Table 2.

**Table 2:** Summary of potential surgical approaches to the superior medullary velum

Surgical approach	Pros	Cons
Transvermmian approach	• Short surgical corridor • Increase degrees of freedom for midline located lesions • Surgeon comfort	• Decrease degrees of freedom for lateral located lesions • Resection of healthy cerebellum • Cerebellar akinetic mutism syndrome • Palatal tremor
Infratentorial supracerebellar approach	• Good maneuverability to both median and lateral lesions located within the superior half of the SVM • Familiar to most neurosurgeons	• Limited access to the inferior half of the SVM • Significant cerebellar retraction to lower seated lesions within the SVM
Posterior interhemispheric approach	• Confortable position for the surgeon with natural gravity-dependent retraction over the ipsilateral occipital lobe • Good exposure for lesions extending from the posterior fossa to the supratentorial compartment via the incisura	• Limited exposure for ipsilateral lesions • Long surgical corridor for contralateral lesions • Close relation with critical venous structures in midline lesions
Tonsillobiventral approach	• Less post-operative pain • Less amount of dissection to access the lateral recess of the fourth ventricle	• Limited exposure of lesions with higher location in the SVM
Uvulotonsillar telovelar approach	• Retraction free exposure of the floor and lateral recesso of the fourth ventricle and the inferior 2/3 of the SVM	• Technically demanding • Intimate arterial (PICA) relationship

Even though telovelar, transvermian and supracerebellar infratentorial are the most common approaches to posterior fossa pathology, different groups described new surgical approaches to lesions within mesencephalum and fourth ventricle [4, 6, 7, 9, 26] exploring the natural fissures and CSF spaces within the posterior fossa. The fissures around the *Tonsils* have been extensively explored, and different names have been given to similar approaches via similar spaces and fissures [4]. These can be divided into two large groups: medial-tonsillar approaches and lateral-tonsillar approaches. The medial-tonsillar approaches include all the surgical corridors in between the tonsil and the uvula and pyramid of the vermis: tonsilouvular, telovelar and cerebellomedullary fissure approaches. The lateral-tonsillar approach includes all the surgical corridors in between the tonsil and the biventral lobule of the cerebellar hemisphere: supratonsillar and uvulobiventral approaches. The criteria underlying the choice of one of these routes lies in the location of the lesion: medial-tonsillar approach to midline (floor of the fourth ventricle) [27] and superior located lesions (tegmentum and aqueductal lesions) [9] and lateral-tonsillar approach to lateral recess [26] and inferior cerebellar peduncle [18].

For the first time, we describe a medial-tonsillar approach to a SMV lesion. This approach is safer for preservation of both SCP and dentate nucleus when compared with lateral-tonsillar approach. The inferior cerebellar peduncle is an anatomic reference to dentate nucleus presentation as it runs anterior to it [1]. This allows a direct corridor to the SMV region with no need for cerebellar retraction or parenchymal resection. As it is appreciated in the DTI study (Fig. 3), the lesion was located medially to the ipsilateral dentatorubrothalamic tract. Therefore, an approach coming from the fourth ventricle is the only available to preserve this crucial pathway within the SCP. When compared with the transvermian approach, it provides a better access to the foramen of Luschka, lateral and supralateral recesses which is crucial for laterally and inferior located lesions in the SMV. When compared with the previous approaches, it has a decreased exposure of the superior half of the SMV, near the aqueduct (> 75% the distance between the obex and the aqueduct). It is technically demanding considering the exposure of the craniovertebral junction and the close relation with the PICA along the exposure and the vertebral artery during the initial bone exposure. Nevertheless, the vascular intimal relationship is the surgical endeavour of the posterior interhemispheric approach as well, which balance both these approaches in term of the vascular risk. Even though the craniocervical junction is exposed and resection of the posterior arch of C1 and a suboccipital craniotomy is performed, the craniovertebral instability is an uncommon event. The risk of hydrocephalus can be minimized by the bilateral exposure the cerebellomesencephalic fissure and the augmentation of the fourth ventricle (communication with the aqueduct and the cisterna magna) [27]. Even though the resection of the tonsils can provide a wider corridor to the lateral (Luschka foramina) and superior (Sylvian aqueduct) structures [15], the preservation of the non-involved anatomical structures should be attempted as they can provide support to the cerebellar retraction if needed and provide anatomical orientation if redo surgery is required.

## Conclusion

The *Tonsils* are key anatomical structures in defining the surgical corridors in posterior fossa pathology located in the four ventricles, brain stem and surrounding structures. The telovelar approach allows a retraction-free and anatomical tract-sparing approach to the SMV region. The retraction or resection of normal anatomical and pathological-free SCP should be avoided in order to minimize the risk of post-operative cerebellar mutism, ataxia, tremor, dysmetria, decomposition of movement in the ipsilateral extremities, nystagmus, hypotonia or palatal tremor, all possible complications of SCP injury.
